# Retinoic Acid and Arsenic for Treating Acute Promyelocytic Leukemia

**DOI:** 10.1371/journal.pmed.0020012

**Published:** 2005-01-25

**Authors:** Guang-Biao Zhou, Wei-Li Zhao, Zhen-Yi Wang, Sai-Juan Chen, Zhu Chen

## Abstract

What were the critical steps in the development of ATRA and arsenic as treatments for APL? Researchers in Shanghai tell the story and look to the future

Acute promyelocytic leukemia (APL) was first identified as a distinct subtype of acute myeloid leukemia in 1957 by Leif Hillestad. It is called M3 in the French–American–British classification, with a variant type referred to as microgranular (M3v in the French–American–British nomenclature) [1]. APL is characterized by three features: (1) the presence of an accumulation of abnormal promyelocytes (see Glossary) that do not differentiate into mature granulocytes, (2) the occurrence of fibrinogenopenia and disseminated intravascular coagulation that is often worsened by chemotherapy, and (3) the presence of the specific chromosomal translocation t(15;17)(q22;q21) (Figure 1).

**Glossary**

**Apoptosis:** A genetically determined process of cell death in which the cell uses specialized cellular machinery to kill itself and is then eliminated by phagocytosis or by shedding.

**Caspase:** A family of cysteine proteases with aspartate specificity that are essential intracellular death effectors.

**Disseminated intravascular coagulation:** A hemorrhagic disorder that occurs following the uncontrolled activation of clotting factors and fibrinolytic enzymes throughout small blood vessels, resulting in depletion of clotting factors and tissue necrosis and bleeding.

**Fibrinogenopenia:** A decrease in concentration of fibrinogen in the blood.

**Granulocyte:** Terminally differentiated myelocyte or polymorphonuclear white blood cell (as a basophil, eosinophil, or neutrophil) with granule-containing cytoplasm.

**Ligand-inducible transcription factors:** Transcription factors that structurally have domains associated with DNA binding and ligand (hormone) recognition. When binding to its specific ligand, the transcription factor initiates a series of conformational changes and interacts efficiently with its specific DNA response element to recruit components of the transcriptional machinery.

**Nuclear receptor superfamily:** One of the most abundant classes of transcriptional regulators including receptors for steroid hormones (e.g., estrogens, glucocorticoids, and vitamin D3), RAs, thyroid hormones, and so on. These transcription factors regulate diverse functions such as homeostasis, reproduction, development, and metabolism in animals.

**Promyelocyte:** Granule-containing cell in bone marrow that is in an intermediate stage of development between myeloblasts and myelocytes and that gives rise to a granulocyte.

**Proteasome:** Proteolytic complex that degrades cytosolic and nuclear proteins.

**Sumoylation:** Post-translational modification of proteins by the small ubiquitin-like modifier SUMO.

**Ubiquitin:** A chiefly eukaryotic protein that when covalently bound to other cellular proteins marks them for proteolytic degradation.

**Figure 1:**
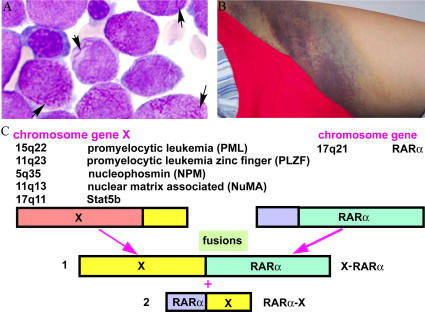
The Three Features of APL The three features of APL are (A) accumulation of abnormal promyelocytes, (B) fibrinogenopenia and disseminated intravascular coagulation, and (C) the chromosomal translocation t(15;17)(q22;q21), the resultant fusion transcripts, and variants.

APL accounts for 10%–15% of all cases of acute myeloid leukemia, with several thousand new cases diagnosed worldwide each year. Before the advent of differentiation therapy, APL was treated with anthracycline-based chemotherapy with a complete remission rate of 60%–76% and a 5-year event-free survival rate of 23%–35% [1,2].

## Differentiation Therapy: From Hypothesis to Practice

Failure to differentiate terminally characterizes most, if not all, cancer cells of every origin. Whether the induction of differentiation could be a treatment strategy for cancers was hotly debated for decades before the advent of differentiation therapy.

**Figure 2:**
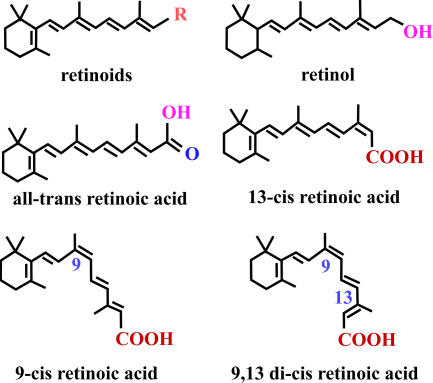
Isomers of RA

An important discovery of the early 1970s was that myeloid leukemic cells could be reprogrammed to resume normal differentiation and to become non-dividing mature granulocytes or macrophages as a result of stimulation by various cytokines [3,4]. Based on this discovery, Leo Sachs at the Weizmann Institute of Science, Rehovot, Israel, hypothesized in 1978 that treatment with agents that induce cancer cells to complete differentiation could be a potential therapeutic option for patients with cancer [5]. In the early 1980s, Breitman and colleagues showed that retinoic acid (RA; Figure 2), a derivative of vitamin A, could induce terminal differentiation of human promyelocytic leukemic cells in vitro [6,7]. But the first clinical reports of using RA showed conflicting results. Some case reports showed beneficial effects of 13-*cis* RA in people with refractory or relapsed APL [8,9,10], but other reports showed that 13-*cis* RA was ineffective in treating APL [11].

Beginning in the early 1980s, the Shanghai Institute of Hematology conducted a series of experiments on differentiation therapy for APL. These experiments showed that all-trans RA (ATRA) could induce terminal differentiation of HL-60, a cell line with promyelocytic features, and fresh leukemic cells from patients with APL. These intriguing results were the impetus for a clinical trial. Twenty-four patients with APL were treated with ATRA (45 to 100 mg/m^2^/day). The result was dramatic: 23 patients (95.8%) went into complete remission (CR) without developing bone marrow hypoplasia or abnormalities of clotting. The remaining one patient achieved CR when chemotherapy was added [12]. Morphological maturation of bone marrow cells was found in all patients studied.

These results were later confirmed by many randomized studies in centers around the world. Further trials showed improved rates of CR, a decrease in severe adverse effects, and lengthening of the duration of remission. Table 1 summarizes the CR rates obtained in most large series of patients. Currently, ATRA combined with anthracycline-based chemotherapy can achieve CR in 90%–95% of patients with APL and overall 5-year disease-free survival in up to 75% of patients [13].

**Table 1:**
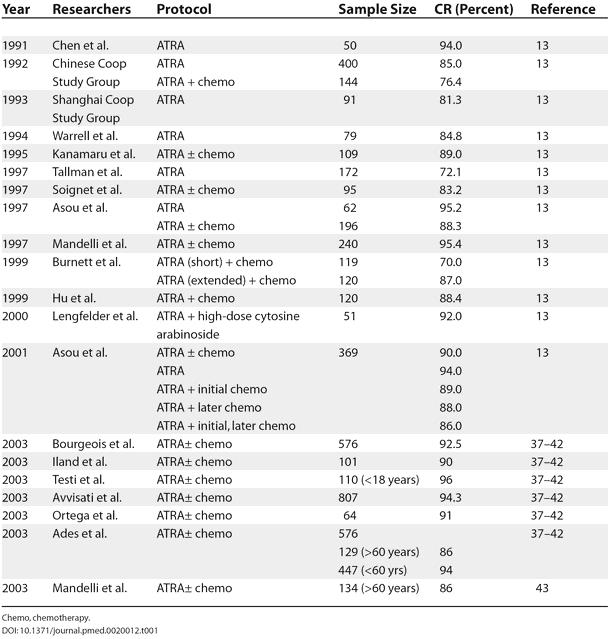
CR Rate in Patients with APL Treated with ATRA (in Series Including More Than 50 Cases)

Chemo, chemotherapy

## Arsenic: “Ancient Remedy Performs New Tricks”

Arsenic is a common, naturally occurring substance that exists in organic and inorganic forms in nature. The organic arsenicals consist of an arsenic atom in its trivalent or pentavalent state linked covalently to a carbon atom. There are three inorganic forms of arsenic: red arsenic (As_4_S_4_, also known as “realgar”), yellow arsenic (As_2_S_3_, also known as “orpiment”), and white arsenic, or arsenic trioxide (As_2_O_3_) [14].

Arsenic was used to treat chronic myelogenous leukemia in the 18th and 19th centuries, but was discarded as a treatment in the early 20th century because of its toxicity and the advent of radiation and cytotoxic chemotherapy. In the early 1970s, a group from Harbin Medical University in China found that intravenous infusions of Ailing-1, a crude solution composed of 1% arsenic trioxide with a trace amount of mercury chloride, induced CR in two-thirds of patients with APL. There was an impressive 30% survival rate after 10 years [15,16]. Pure arsenic trioxide at 0.16 mg/kg/day for 28–54 days was shown to induce CR in 14 out of 15 (93.3%) patients with relapsed APL [17]. Tetra-arsenic tetra-sulfide was also reported to be effective in APL treatment [18].

Since 1996, a large number of reports have shown that arsenic compounds induce a CR in 85% to 90% of patients with both newly diagnosed and relapsed APL [13]. Furthermore, after CR is achieved by arsenic compounds, a molecular remission (i.e., negative for promyelocytic leukemia RA receptor a [PML-RARa] transcript detected by reverse transcriptase polymerase chain reaction) is obtainable either with arsenic compounds or with ATRA and chemotherapy as consolidation treatment. It seems likely that arsenic compounds appropriately used in post-remission therapy could prevent recurrence and achieve a longer survival time [13,18].

Studies have shown that arsenic trioxide exerts dose-dependent dual effects on APL cells—it induces apoptosis (programmed cell death) preferentially at relatively high concentrations (0.5 × 10^−6^ to 2 × 10^−6^ M) and induces partial differentiation at low concentrations (0.1 × 10^−6^ to 0.5 × 10^−6^ M). The rapid modulation and degradation of the PML-RARa oncoprotein by arsenic trioxide could contribute to these two effects [19].

## How Do ATRA and Arsenic Work at the Molecular Level?

To understand how ATRA and arsenic compounds act at the molecular level in treating APL, it is first important to understand the role of the PML-RARa fusion protein in the pathogenesis of APL.

**Figure 3:**
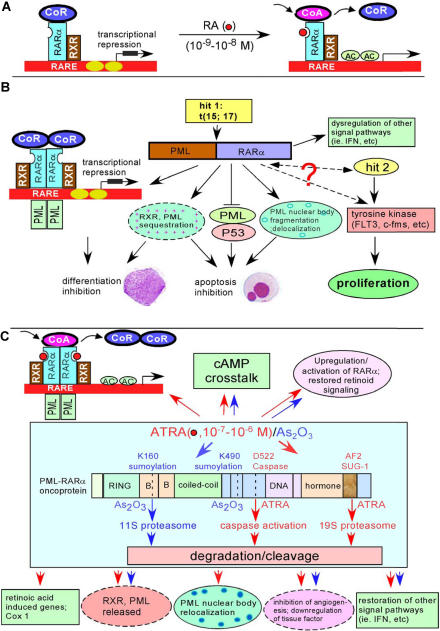
Leukemogenic Effects of PML-RARá and Mechanisms of ATRA/Arsenic Trioxide in the Treatment of APL (A) In the absence of RA, RARα/RXR heterodimers recruit the transcription corepressor (CoR), which mediates transcriptional silencing by mechanisms that include direct inhibition of the basal transcription machinery and recruitment of chromatin-modifying enzymes. Chromatin modification includes histone deacetylation, which leads to a compact chromatin structure that impairs the access of transcriptional activators. In the presence of physiological concentrations (10^−9^–10^−8^ M) of RA, the transcription corepressor is released and the coactivator is recruited to the RARα/RXR heterodimer, resulting in histone acetylation (AC) and overcoming of the transcription blockage. (B) PML-RARα fusion protein binds to RARα target genes either on its own or with RXR and then recruits corepressors, leading to transcriptional repression and myeloid differentiation inhibition. PML-RARα oncoprotein sequesters the normal RXR and PML, inhibits the PML/P53 apoptotic pathway, and delocalizes PML and other proteins from the nuclear body. PML-RARα also may affect interferon (IFN) and other signal pathways. Abnormalities in protein tyrosine kinases (e.g., FLT3, c-fms) may collaborate with PML-RARα to cause APL. (C) In the presence of pharmacological doses of ATRA or arsenic trioxide, the PML-RARα fusion is degraded in ways that are dependent on caspases and proteasomes. The degradation of PML-RARα may lead to derepression of transcription suppression and restoration of PML nuclear body structure. The blockade of other signaling pathways is also released, and the anti-apoptotic effect of PML-RARα is lost. ATRA also induces cyclic AMP (cAMP), which reverses the silencing of RXR, induces the expression of RA-induced genes and cyclooxygenase 1 (Cox 1), inhibits angiogenesis, and downregulates tissue factor. Subsequently, ATRA induces terminal cell differentiation, while arsenic trioxide induces partial differentiation and/or apoptosis of APL cells. The effects of ATRA and arsenic trioxide are indicated with red and blue arrows, respectively. AF2, the ligand-dependent transcriptional activation domain contained within the C-terminal E domain of RARα; D522, aspartate at residue 522; K160, lysine at residue 160; K490, lysine at residue 490; RARE, retinoic acid response element; SUG-1, a component of proteasome 19S complex that can bind with the activated AF2 domain of RARα.

Retinoids that are crucial for normal myeloid differentiation act via RA receptors (RARs) and retinoid X receptors (RXRs). These belong to the steroid/thyroid/retinoid nuclear receptor superfamily of ligand-inducible transcription factors. Both RAR and RXR families consist of three subtypes: α, β, and γ [20]. RARα forms a heterodimer with RXR and binds to RA response element to control the expression of target genes in the presence of physiological concentrations (10^−9^–10^−8^ M) of retinoids (Figure 3A).

More than 95% of patients with APL have the t(15;17)(q22;q21) translocation. This results in the fusion of the RARα gene on 17q21 and the promyelocytic leukemia (PML) gene on 15q22, which generates a PML-RARaα fusion transcript [21,22]. Variant translocations can also be detected in APL. The PML-RARα chimeric protein acts as a dominant negative mutant over wild-type RARα. The chimeric protein prevents activation of key target genes required for normal myeloid differentiation by sequestering RXR and other RARa cofactors and inhibiting normal RARα functions. The PML-RARα oncoprotein binds to RAR target genes either on its own or with RXR and then recruits histone deacetlyase complexes, which act as repressors of transcription.

PML-RARa may affect transcription in other pathways including those in which the transcription factor AP1 and interferon-responsive factors are involved. PML-RARα also binds to promyelocytic leukemia zinc finger (PLZF) protein and potentially affects its functions (e.g., growth suppression and transcription repression; control of developmental programs and differentiation) [20]. In addition, PML-RARα prevents apoptosis through delocalization of PML and other proteins from the nuclear body. Finally, PML-RARα may cooperate with activated mutations in protein tyrosine kinases, such as FLT3 [23], which confer proliferative and/or survival advantage to hematopoietic stem/progenitor cells. Whether PML-RARα affects the protein tyrosine kinase activity directly or indirectly is unclear. All these interactions of PML-RARα could be involved in the leukemogenesis of APL (Figure 3B).

ATRA and arsenic trioxide degrade and cleave the PML-RARα oncoprotein. Although we now have a good understanding of the molecular mechanisms underlying ATRA in differentiation therapy for APL, these mechanisms were shown long after the identification of the efficacy of this drug in treating the disease. Now it is well established that pharmacological concentrations of ATRA (10^−7^–10^−6^ M) exert their effects through targeting the PML-RARα oncoprotein, triggering both a change in configuration and degradation of the oncoprotein and the activation of transcription, leading to differentiation. Cleavage of the PML-RARα fusion protein by caspases at residue D522 has been shown in APL cells induced to differentiate by ATRA [24].

Further dissecting of the pathways involved in PML-RARα catabolism led to the discovery of ubiquitin/proteasome-mediated degradation of PML-RARα and RARα, which was dependent on the binding of SUG-1 in the AF2 transactivation domain of RARα with 19S proteasome [25,26]. In contrast to ATRA, which targets the RARα moiety of the fusion, arsenic targets the PML moiety of PML-RARα, through a still unclear mechanism, and causes PML to localize to the nuclear matrix and become sumoylated. Sumoylation at K160 is necessary for 11S proteasome recruitment and arsenic-trioxide-induced degradation, whereas sumoylation at K490 is needed for nuclear localization [27,28]. These results provide a striking similarity in the effect of these two otherwise unrelated agents (Figure 3C).

The final result of treatment with ATRA and arsenic trioxide is the degradation of the PML-RARa oncoprotein, which results in restoration of normal retinoid signaling. RXR and PML sequestration is abrogated, and PML nuclear body is restored. The corepressor is released and the coactivator is recruited and bound with RARα; thus, the transcription of target genes is derepressed. ATRA also induces cyclic AMP, a differentiation enhancer that boosts transcriptional activation, reverses the silencing of the transactivating function of RXR, and restores ATRA-triggered differentiation in mutant ATRA-resistant APL cells [29]. Additionally, ATRA induces the expression of RA-induced genes [30] and cyclooxygenase 1 [31], inhibits angiogenesis [32], downregulates the expression of tissue factor [33], and restores other signal pathways (e.g., the interferon pathway). Consequently, the abnormal promyelocytes differentiate and die through programmed cell death (Figure 3C).

## Combining ATRA and Arsenic: A Cure for APL?

Since ATRA and arsenic trioxide degrade the PML-RARa oncoprotein via different pathways, and since studies in animal models have shown synergic effects of both drugs in prolonging survival or even eliminating the disease [34,35], the group at the Shanghai Institute of Hematology hypothesized that the combination of the two drugs might synergize in treating APL. To test this, 61 patients newly diagnosed with APL were randomized into three treatment groups: ATRA, arsenic trioxide, or a combination of the two drugs [36]. Although CR rates in all three groups were high (>90%), the time to achieve CR differed significantly—the time was shortest in patients treated with the combination. The disease burden (as reflected by fold change of PML-RARα transcripts at CR) decreased significantly more with combined therapy than with ATRA or arsenic trioxide monotherapy (*p* < 0.01), and this difference persisted after consolidation therapy (*p* < 0.05). Notably, all 20 patients in the combination group remained in CR whereas seven of 37 cases treated with monotherapy relapsed (*p* < 0.05) after a follow-up of 8–30 months (median, 18 months).

It seems that a combination of ATRA and arsenic trioxide for remission and maintenance treatment of APL produces better results than either of the two drugs used alone, in terms of the time required to achieve CR and the length of disease-free survival. We hope that the use of three treatments—ATRA, arsenic trioxide, and chemotherapy—will ultimately make APL a curable human acute myeloid leukemia [36].

## Conclusion

The story of ATRA in the treatment of APL shows that by targeting the molecules critical to the pathogenesis of certain diseases, cells can be induced to return to normal. Differentiation therapy is therefore a practical method of treating human cancer that has shown consistent effectiveness in trials. The clarification of the underlying molecular abnormalities of APL is an example of the benefits of a close collaboration between bench and bedside, and is necessary for our understanding of the mechanisms of ATRA in differentiation therapy. It is clearly important to elucidate the molecular and cellular basis of a particular cancer if we are to further develop mechanism-based target therapies.

The sequencing of the human genome and ongoing functional genomic research are now accelerating the dissection of disease mechanisms and identification of therapeutic targets. This in turn may facilitate the screening of promising treatments. What we learn from developing curative treatment approaches to APL may help to conquer other types of leukemias and cancers.

## Abbreviations

APL: acute promyelocytic leukemia
ATRA: all-trans retinoic acid
CR: complete remission
PML: promyelocytic leukemia
PML-RARá: promyelocytic leukemia retinoic acid receptor á
RA: retinoic acid
RAR: retinoic acid receptor
